# The expansion of culturable opportunistic pathogens and antibiotic resistance in mouse gut following antibiotic exposure

**DOI:** 10.1128/spectrum.01104-25

**Published:** 2025-12-05

**Authors:** Bianfang Wang, Jian Zhang, Yuqing Sun, Xin Wei, Wenli Lai, Tingting Guo, Chengzhang Fu, Youming Zhang, Hai Xu, Ling Li, Mingyu Wang

**Affiliations:** 1 State Key Laboratory of Microbial Technology, School of Life Sciences, Shandong University 214177 , Qingdao, Shandong, China; 2 Workgroup Genome Mining for Secondary Metabolites, Helmholtz Institute for Pharmaceutical Research Saarland (HIPS), Helmholtz Centre for Infection Research, Saarland University 443745, Saarbrücken, Germany; 3 Helmholtz International Lab for Anti-Infectives, Helmholtz Center for Infection Research 28336, Braunschweig, Germany; Peking University People's Hospital, Beijing, China

**Keywords:** gut microbiota, Enterobacteriaceae, *Enterococcus*, *Lactobacillus*, imipenem, ampicillin, culturing-dependent analysis

## Abstract

Antibiotics significantly change gut microbiota, leading to dysbiosis and changes in bacterial community composition, which further results in a series of dysfunctions with severe health consequences. This work employed both culture-dependent and -independent methods to examine antibiotic-induced changes in gut microbiota at higher resolution using mouse models. By following bacterial changes and antibiotic resistance daily before antibiotic exposure, after antibiotic exposure, and during recovery, several significant novel findings were made. Ampicillin (AMP) and imipenem (IPM) treatment led to an acute, exponential, but temporary bloom in absolute abundances of live opportunistic pathogens Enterobacteriaceae and *Enterococcus*, while not affecting potentially beneficial *Lactobacillus*. The absolute abundances of antibiotic-resistant opportunistic pathogens followed a similar pattern. Impact on antibiotic resistance, on the other hand, is significant and persistent. Nearly, all Enterobacteriaceae performed AMP-resistant at the end of treatment with AMP, and almost 10% of *Enterococcus* became IPM-resistant following treatment with IPM. Further analysis of the genomes of isolated bacteria suggested that the former was due to replacement of Enterobacteriaceae strains while the latter is not. These studies show that antibiotic exposure led to expansion of gastrointestinal pathogens and their resistance to antibiotics, increasing risks for bacterial infections, contrary to the traditional anti-infection functions of antibiotics.

**IMPORTANCE:**

The antibiotic therapy is the primary method for treating and preventing bacterial infections. However, the consumption of antibiotics may also cause collateral damage in the normal microbial flora in human body, leading to dysbiosis with unwanted consequences. This impact was previously studied mostly with sequencing-based methods, which did not distinguish between live and dead cells, and did not provide absolute quantitative data, limiting the physiological relevance of discoveries. This work expands the methods in studying the side effects of antibiotic therapy by including culturing methods that measure absolute quantities of live bacteria. Surprising findings were made that antibiotic uptake can lead to temporary exponential bloom of opportunistic pathogens in mouse gut, and a permanent change of antimicrobial resistance in these pathogens. These findings expand our knowledge on how antibiotic therapy can affect our health and urges caution on casual antibiotic usage.

## INTRODUCTION

Antibiotics, arguably the most important medicine in the last century, rank among the most frequently consumed pharmaceuticals with prescription, or as over-the-counter drugs. An average of 14.1 Defined Daily Doses (DDDs) of antibiotics were consumed for every 1,000 inhabitants globally from 2000 to 2018 (1). The highest level of antibiotic consumption reached 45.9 DDDs/1,000 inhabitants, equivalent to over 4% of the population taking antibiotics every day. While antibiotics are nearly irreplaceable for the treatment and prevention of bacterial infection, side effects and association with unexpected outcomes have been widely reported. Besides widely known stress to liver and kidney, antibiotic uptake was known to impair immunity against viruses leading to viral infections (2), aggravate allergic syndromes (3), decrease vaccine efficacies (4), reduce responses of anticancer immunotherapy (5), induce asthma and elevated body mass indices (6), promote diabetes progress (7), and lead to antibiotic-associated diarrhea (AAD) among 17.5% of the patients (8).

Ironically, the bactericidal effects of antibiotics can sometimes contribute to subsequent bacterial infections. Colonization resistance, the mechanism by which a balanced gut microbiota confers protection against pathogens, is damaged by antibiotics (9). It was shown that the biggest risk factor for *Clostridium difficile* infection (CDI) is antibiotic uptake (10). This toxin-producing pathogen with strong antibiotic resistance can lead to long-lasting diarrhea (11). Modulation of host regulatory mechanisms was also found to be involved. Antibiotic treatment impairs lipopolysaccharide and flagellin-sustained expression of the C-type lectin RegIII-γ, rendering mouse models susceptible to VRE infection (12–15). Gram-positive-targeting antibiotics were shown to damage segmented filamentous bacteria (SFB), inducers of Th17 differentiation, thereby resulting in increased susceptibility to bacterial infections (16). Perinatal antibiotic exposure alters neutrophil numbers and increases the risk of sepsis caused by Enterobacteriaceae in neonates (17). In a large surveillance involving 5.4 million people from 516 hospitals, uptake of antibiotics was found to lead to increased risks for sepsis (18).

The ultimate reason why antibiotic administration results in the above mentioned adverse effects is its disruption of the stability of bacterial communities, particularly in the gastrointestinal tract. Recent developments in metagenomic technologies have accelerated the analysis of these perturbations. Despite the high heterogeneity between studies and subjects, a shared observation is the reduction of diversity as a result of antibiotic treatment (19, 20). In many reports, relative abundances of Enterobacteriaceae and Enterococcaceae containing many opportunistic pathogens increased (21–30), whereas relative abundances of beneficial bacteria *Bifidobacterium* and *Lactobacillus* dropped (22–24, 30). Many of these changes are temporary and disappear after stopping antibiotics (11, 31), but microbiomic “scars,” or permanent changes, have also been observed (32, 33). These investigations tremendously improved our understanding of how antibiotics modulate gut microbiota and identified changes that are apparently detrimental. However, from a microbiological point of view, they do not fully represent functional changes as only relative abundances of representative DNA sequences, rather than viable cells, were quantified.

Consumption of antibiotics may also lead to elevated antimicrobial resistance (AMR) in the gastrointestinal tract and other parts of the human body. AMR poses significant threats to human health. It was estimated that 1.3 million lives were lost globally due to AMR in 2019 (34), and it is well documented that AMR development is correlated with antibiotic usage from population surveillances (35). However, the evolution and dissemination of AMR mechanisms are not entirely antibiotic-dependent. Reports have shown that antibiotic resistance genes (ARGs) were prevalent in an 11th-century A. D. mummy (36), 30,000-year-old Beringian permafrost sediment (37), isolated cave in New Mexico (38), and pristine soil in Antarctica (39). These environments were never touched by humans, let alone modern antibiotics. The evolution of resistance in response to antibiotics, therefore, is quite complicated. Controversial reports were available, with evidence that antibiotic uptake promotes antibiotic-resistant bacteria (ARB) (40, 41) and ARGs in the gastrointestinal tract (32, 33, 42–44), while findings that these responses were lacking, temporary or even opposite also exist (21, 45). It was also observed that the evolution of resistance of only a small fraction of bacteria results in improved overall resistance and stability of the whole bacterial community (46, 47).

This work aims to inspect the impact of antibiotics on gut microbiota from a non-metagenomic perspective: by analyzing changes in live bacterial cells. This perspective provides additional information on the absolute abundances of bacteria of interest and ensures that conclusions are physiologically relevant. We believe this culturing-dependent approach better describes changes in gut microbiota functions, as metagenomic methods do not distinguish between live and dead cells, and cannot associate antibiotic resistance properties with specific bacterial groups. After all, it is the live cells that are functional, and the absolute numbers of bacteria are more meaningful than relative proportions when their association to human health is discussed. In this study, culture-dependent absolute quantification revealed transient blooms of viable Enterobacteriaceae and *Enterococcus*, as well as persistent resistance increases driven by different mechanisms, insights that would not be captured by relative sequencing data alone.

## RESULTS

### Promotion of total bacterial biomass in mouse feces following antibiotic exposure

**Fig 1:**
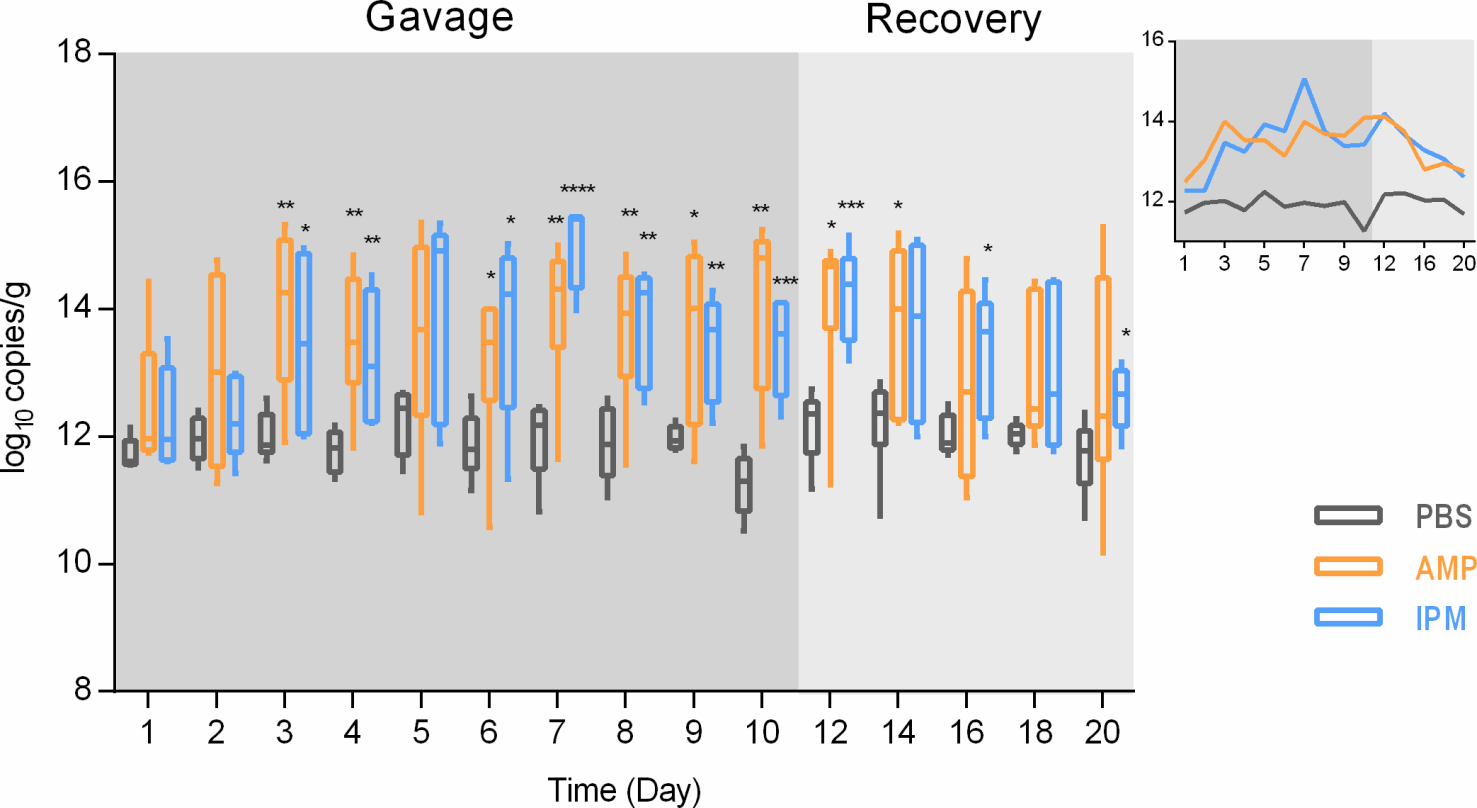
Bacterial biomass levels in murine fecal samples. Biomass was represented by 16S rDNA copies per gram of feces. Side figure is an abbreviated plot of 16S rDNA mean values. Axes and legends are the same as main figure. *, *P* < 0.05; **, *P* < 0.01; ***, *P* < 0.001; ****, *P* < 0.0001.

Thirty mice were subjected to exposure to ampicillin (10), imipenem (10), and PBS (10) by gavage for 10 days, followed by 10 days of recovery. No significant differences in body weight could be observed following exposure, suggesting generally unchanged overall health (Fig. S1). Fecal samples as representatives of gut microbiota were analyzed for 16S rDNA levels, to reflect total bacterial biomass (live and dead cells combined) in gastrointestinal tracts. It was observed that feeding mice with PBS did not change bacterial biomass in fecal samples (Fig. 1). However, feeding mice with either AMP or IPM led to significant overall increase of bacteria levels. Bacterial biomass was found to steadily increase following initiation of gavage and peaked after 7 days of continuous gavage (2.03 log fold for AMP and 3.11 log fold for IPM). Biomass levels started to decline as gavage stopped and resumed to early-gavage levels after 10 days of recovery.

### Increase of Enterobacteriaceae relative abundances following antibiotic exposure

**Fig 2:**
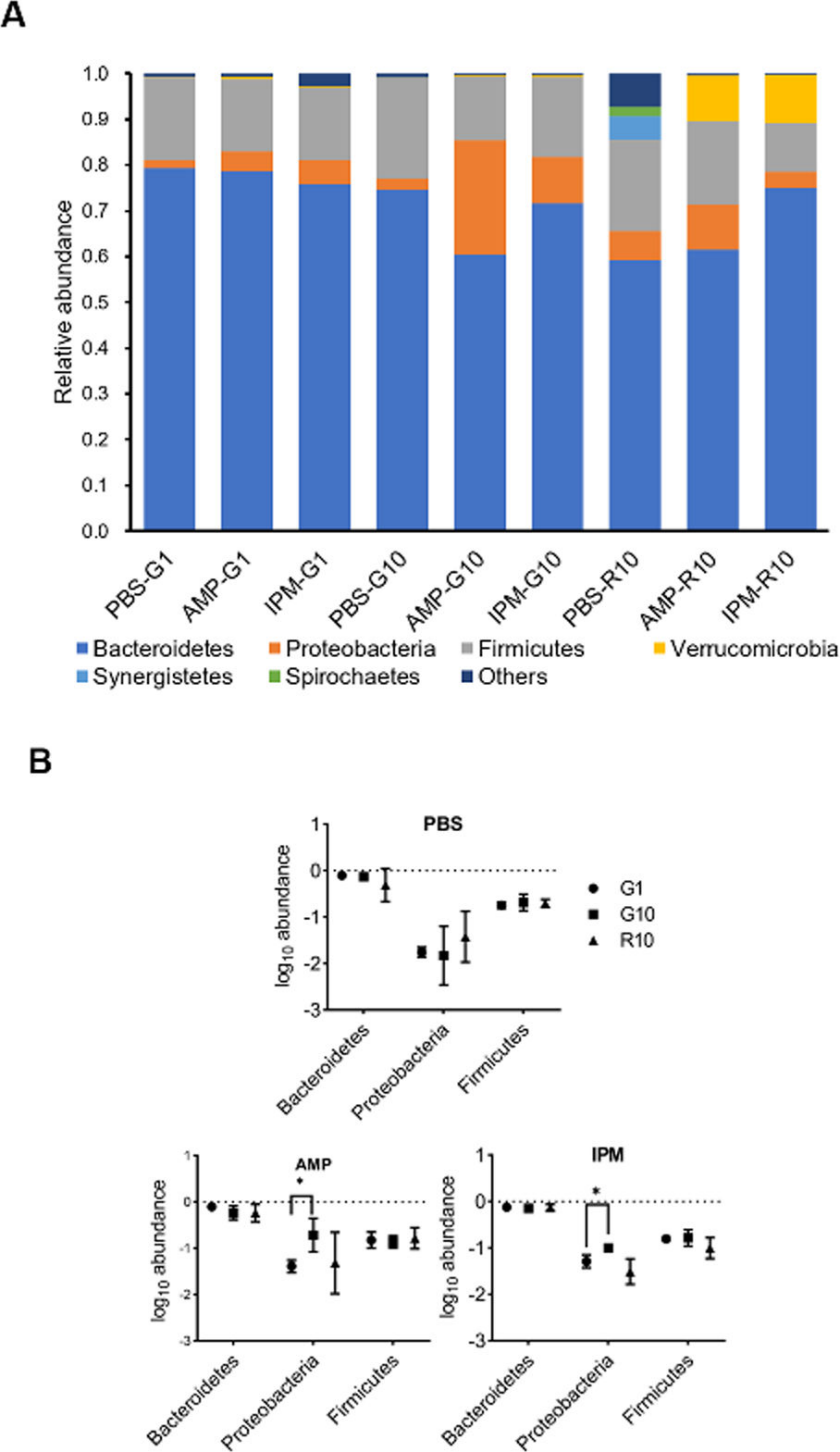
Bacterial community compositions in fecal samples. (**A**) Average bacterial community composition at the phylum level. (**B**) Comparison of Bacteroides, Proteobacteria, and Firmicutes abundances in fecal samples. G1, first day after gavage; G10, 10th day after gavage; R10, 10th day after recovery. *, *P* < 0.05.

The bacterial community compositions in fecal samples following antibiotic exposure were investigated by high throughput 16S rDNA amplicon sequencing (Fig. 2). While the fecal microbiomes were highly similar at the start of gavage of either PBS, AMP, or IPM, an increase of Proteobacteria in AMP- and IPM-exposed groups was found at the 10th day of gavage (Fig. 2A). By plotting and comparing relative bacterial abundances, an increase in Proteobacteria in fecal samples at the end of antibiotic exposure is significant (*P* = 0.038 for AMP, *t* = 3.039, *n* = 3; *P* = 0.033 for IPM, *t* = 3.194, *n* = 3; Fig. 2B). At the family level, the relative abundance of Enterobacteriaceae, which belong to the Proteobacteria phylum, was significantly increased after AMP and IPM exposure (*P* = 0.024, *t* = 3.535, *n* = 3; *P* = 0.027, *t* = 3.409, *n* = 3; Fig. S2). Detailed analysis revealed that the abundance of Enterobacteriaceae increased from 2.88% ± 0.53% to 23.49% ± 22.11% after AMP exposure and from 1.46% ± 1.18% to 6.08% ± 0.21% after IPM exposure. Further LefSe analysis supported increase of Enterobacteriaceae after AMP exposure (Fig. S3). Considering the high proportion of Enterobacteriaceae after antibiotic exposure, we suspected that antibiotics could lead to the increase of this important enteric bacteria family to which many opportunistic pathogens including *Escherichia coli*, *Klebsiella pneumoniae*, and *Salmonella enterica* belong, and this increase may significantly contribute to the increase of overall bacterial biomass after antibiotic exposure. It needs to be noted here that the increase of Enterobacteriaceae proportion diminished at the end of recovery, in consistency with the disappearance of bacterial biomass increase at the end of recovery. This finding is in agreement with previous studies that showed expansion of Enterobacteriaceae in gut microbiota following antibiotic treatment (29).

### Strong acute promotion of culturable Enterobacteriaceae and *Enterococcus* levels, but not *Lactobacillus* levels following antibiotic exposure

Although bacterial community analysis of antibiotic-exposed murine fecal samples suggests an increase in Enterobacteriaceae, the 16S rDNA amplicon sequencing method is a culture-independent approach, which only reports relative abundances and does not reflect viable bacteria in samples. Both live and dead cells contain 16S rDNA and are counted in this analysis. Therefore, it cannot accurately describe functional microbiomes present in samples.

Accordingly, we further applied culture-based approaches that only consider live cells to determine bacterial community changes after antibiotic exposure. In this experiment, in addition to Enterobacteriaceae, the common gastrointestinal opportunistic pathogen *Enterococcus* and the key beneficial bacterium *Lactobacillus* were also included. From 16S rDNA amplicon sequencing data, the relative abundances of Enterobacteriaceae, *Lactobacillus*, and *Enterococcus* were, respectively, 4.86%, 1.51%, and 0.78%, respectively. This suggests that Enterobacteriaceae is the largest group of the Proteobacteria phylum (average abundance 7.62%), and *Lactobacillus*/*Enterococcus* combined are the most important non-Clostridiales Firmicutes (Firmicutes average abundance 16.92%, Clostridiales order average abundance 12.98%, non-Clostridiales Firmicutes 3.94%, *Lactobacillus*/*Enterococcus* combined 2.29%). Therefore, these three groups of bacteria serve as good representatives of murine fecal and gastrointestinal bacterial communities and are functionally relevant due to their important pathogenic or beneficial roles.

**Fig 3:**
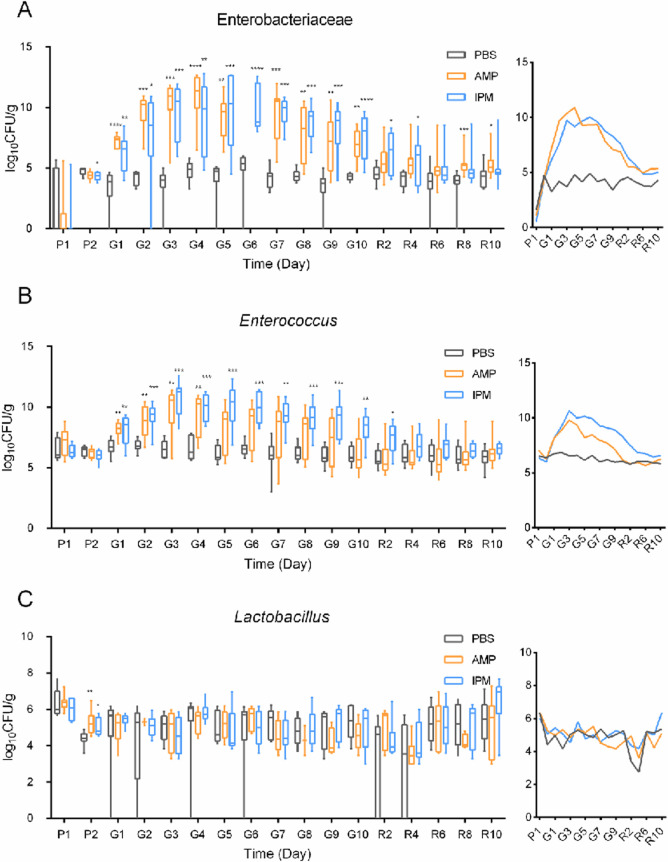
Bacterial levels in murine fecal samples. Bacterial levels were documented in CFU per gram of feces. (**A**) Enterobacteriaceae levels. (**B**) *Enterococcus* levels. (**C**) *Lactobacillus* levels. Gray bars indicate fecal samples of mice fed with PBS, Orange bars indicate fecal samples of mice fed with ampicillin, blue bars indicate fecal samples of mice fed with imipenem. Side figures are abbreviated line plot of CFU means. Axes and color legends are the same as main figures. Statistical analysis was performed with Kruskal-Wallis test. *, *P* < 0.05; **, *P* < 0.01; ***, *P* < 0.001; ****, *P* < 0.0001. P1 and P2, day 1 and day 2 before gavage; G1–G10, day 1 to day 10 after gavage; R2–R10, day 2 to day 10 after recovery.

The culturable bacterial levels in murine fecal samples (in CFU/g feces) were measured with selective agar plates for Enterobacteriaceae, *Enterococcus*, and *Lactobacillus*. As shown in Fig. 3, feeding PBS led to no changes of culturable bacterial levels. Feeding mice with both AMP and IPM led to substantial and significant increases of Enterobacteriaceae (Fig. 3A) and *Enterococcus* (Fig. 3B), whereas no change was observed for *Lactobacillus* (Fig. 3C). For Enterobacteriaceae, AMP treatment resulted in the largest increase on day 7 of gavage, by 4.95 log units, whereas IPM treatment led to the largest increase on day 6 of gavage, with an increase of 6.09 log units. For *Enterococcus*, AMP treatment led to the largest increase on day 3 of gavage, by 2.73 log units, while IPM treatment led to the largest increase on day 3 of gavage, with an increase of 4.29 log units. The increases of Enterobacteriaceae and *Enterococcus* gradually diminished toward the end of gavage, and disappeared at the end of recovery, similarly to what was observed with 16S rDNA amplicon sequencing. These results clearly showed a temporally restricted antibiotic-induced bloom of viable pathogenic bacteria and a lack of response of viable beneficial *Lactobacillus*. Unlike previously reported recovery of gut microbiota that took place after the cessation of antibiotics (21), pathogenic bacteria levels peaked 3–4 days after treatment initiation and started declining during exposure (Fig. 3). Although carrying capacity, typically around 10^10^ CFU/g feces in germ-free mice (48), may partly explain the decline observed during AMP and IPM treatment, our results suggest that this factor alone cannot account for the dynamics. This phenomenon is more consistent with the resilience of gut microbiota to perturbation and abilities to self-heal despite constant exposure to stress.

### Persistent increase of antibiotic resistance in pathogens induced by antibiotics

**Fig 4:**
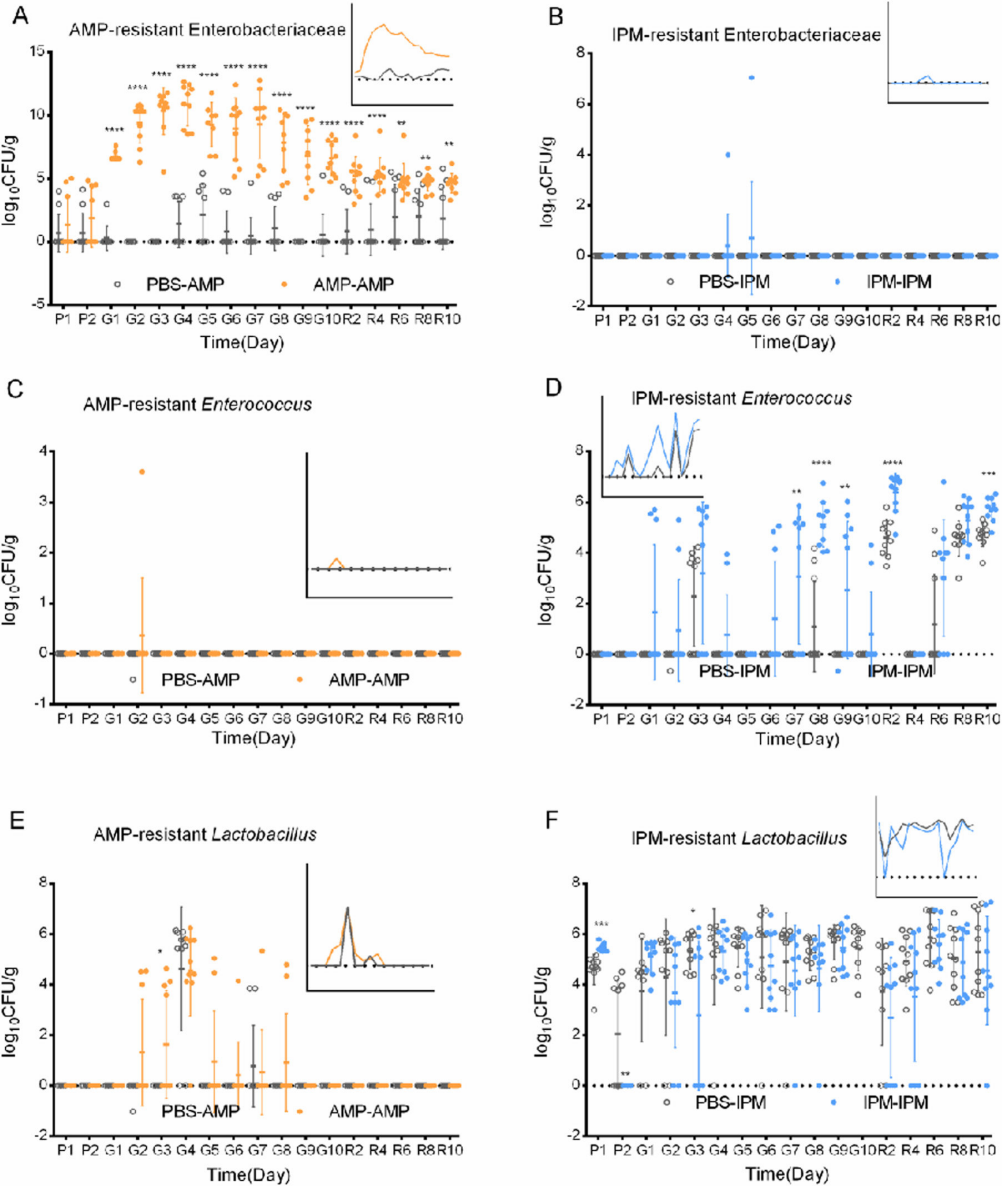
Antibiotic resistant bacteria levels in murine fecal samples. Bacterial levels were documented in CFU per gram of feces. (**A**) AMP-resistant Enterobacteriaceae levels. (**B**) IPM-resistant Enterobacteriaceae levels. (**C**) AMP-resistant *Enterococcus* levels. (**D**) IPM-resistant *Enterococcus* levels. (**E**) AMP-resistant *Lactobacillus* levels. (**F**) IPM-resistant *Lactobacillus* levels. Side figures are abbreviated line plot of CFU means. Axes and color legends are the same as main figures. PBS-AMP, AMP-resistant bacteria from PBS-fed mice; AMP-AMP, AMP-resistant bacteria from AMP-fed mice; PBS-IPM, IPM-resistant bacteria from PBS-fed mice; IPM-IPM, IPM-resistant bacteria from IPM-fed mice. *, *P* < 0.05; **, *P* < 0.01; ***, *P* < 0.001; ****, *P* < 0.0001. P1 and P2, day 1 and day 2 before gavage; G1–G10, day 1 to day 10 after gavage; R2–R10, day 2 to day 10 after recovery. Error bar, standard deviation.

The presence of AMP- and IPM-resistant Enterobacteriaceae, *Enterococcus*, and *Lactobacillus* was investigated in AMP- and IPM-exposed murine fecal samples, and compared to PBS-exposed samples (Fig. 4), in order to understand the impact of antibiotic treatment to antibiotic resistance development in gut microbiota.

**Fig 5:**
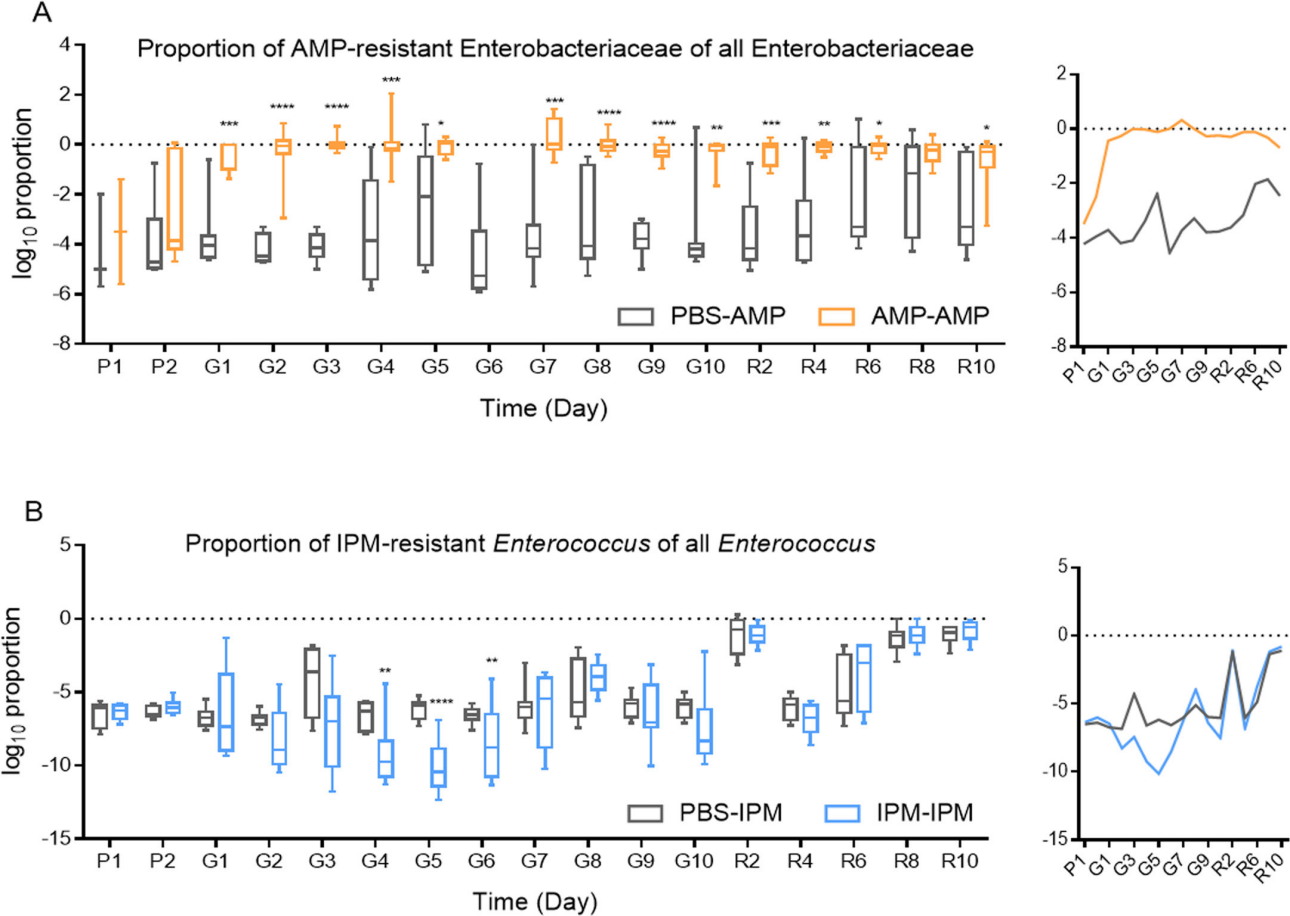
Proportion of antibiotic-resistant bacteria. (**A**) proportion of AMP-resistant Enterobacteriaceae of all Enterobacteriaceae. (**B**) proportion of IPM-resistant *Enterococcus* of all *Enterococcus*. Side figures are abbreviated line plot of proportion means. Axes and color legends are the same as main figures. PBS-AMP, AMP-resistant bacteria from PBS-fed mice; AMP-AMP, AMP-resistant bacteria from AMP-fed mice; PBS-IPM, IPM-resistant bacteria from PBS-fed mice; IPM-IPM, IPM-resistant bacteria from IPM-fed mice. At every time point, the Mann-Whitney test was used for significance correction. *, *P* < 0.05; **, *P* < 0.01; ***, *P* < 0.001; ****, *P* < 0.0001. P1 and P2, day 1 and day 2 before gavage; G1–G10, day 1 to day 10 after gavage; R2–R10, day 2 to day 10 after recovery.

A very low level of AMP- and IPM-resistant Enterobacteriaceae was found in PBS-exposed samples (Fig. 4A and B): average log_10_ CFU/g for AMP-resistant Enterobacteriaceae is 0.93, whereas no IPM-resistant Enterobacteriaceae was found. Gavage of PBS did not lead to increase in antibiotic-resistant Enterobacteriaceae. Gavage of IPM did not lead to a significant increase in IPM-resistant Enterobacteriaceae either, although in two samples during gavage IPM-resistant Enterobacteriaceae was found. Gavage of AMP, on the other hand, led to a strong increase in AMP-resistant Enterobacteriaceae, peaking at the fourth day of gavage, with log_10_ CFU/g = 10.87. This is a strong increase over PBS-exposed samples with a log factor of 9.46. Intriguingly, like total bacterial biomass and culturable Enterobacteriaceae levels, the level of AMP-resistant Enterobacteriaceae declined toward the end of exposure, and kept declining at the end of recovery, but did not return to pre-gavage levels. From the perspective of the proportion of AMP-resistant Enterobacteriaceae among all culturable Enterobacteriaceae, exposure to AMP rapidly, significantly, and substantially increased the proportion (two-way ANOVA, *P* < 0.0001, *F* = 191.7, df = 18, *n* = 10, Fig. 5A). In fact, no significant difference was found in the levels of total Enterobacteriaceae and AMP-resistant Enterobacteriaceae in AMP-exposed murine fecal samples (two-tailed paired *t*-tests, *P* = 0.083, *t* = 1.754, *n* = 84), suggesting nearly all Enterobacteriaceae were AMP-resistant. Therefore, the strong increase in AMP-resistant Enterobacteriaceae is due to both the expansion of Enterobacteriaceae, which peaked 3–4 days after exposure, and the higher proportion of AMP-resistant cells within them. The recovery of AMP-resistant Enterobacteriaceae was the result of reduced Enterobacteriaceae expansion, while AMP resistance persisted and did not decline toward the end of recovery (Fig. 4A and 5A).

No AMP-resistant *Enterococcus* was found in fecal samples collected from PBS-exposed mice. Exposure to AMP did not significantly increase the levels of AMP-resistant *Enterococcus* (Fig. 4C). However, both gavage with PBS and IPM led to a substantial and sustained increase in IPM-resistant *Enterococcus* (Fig. 4D; Fig. S4). The proportion of IPM-resistant *Enterococcus* also substantially increased for both PBS- and IPM-fed mice on the time scale (Fig. 5B). Unlike AMP-resistant Enterobacteriaceae in PBS- and AMP-exposed mice, gavage of PBS and IPM did not lead to pronounced different changes in IPM resistance development for Enterobacteriaceae.

No AMP-resistant *Lactobacillus* were found before gavage of either PBS or AMP (Fig. 4E). Gavage with both PBS and AMP led to an increase in AMP-resistant *Lactobacillus*, but no significant difference was found between PBS and AMP exposure (Fig. 4E, two-way ANOVA, *P* = 0.146, df = 18, *F* = 2.312, *n* = 10 for AMP). This increase disappeared during recovery, suggesting the increase in AMP-resistant *Lactobacillus* was due to gavage. IPM-resistant *Lactobacillus* was present prior to gavage of PBS or IPM at a level of 10^4^-10^6^ CFU/g. These numbers remained largely unchanged during gavage with PBS or IPM (Fig. 4F), possibly because a large portion of *Lactobacillus* were intrinsically IPM-resistant. Notably, IPM did not change IPM-resistant *Lactobacillus* amounts during gavage (two-way ANOVA, *P* = 0.45, df = 18, *F* = 0.5956, *n* = 10) or recovery (two-way ANOVA, *P* = 0.289, df = 18, *F* = 1.195, *n* = 10).

**Fig 6:**
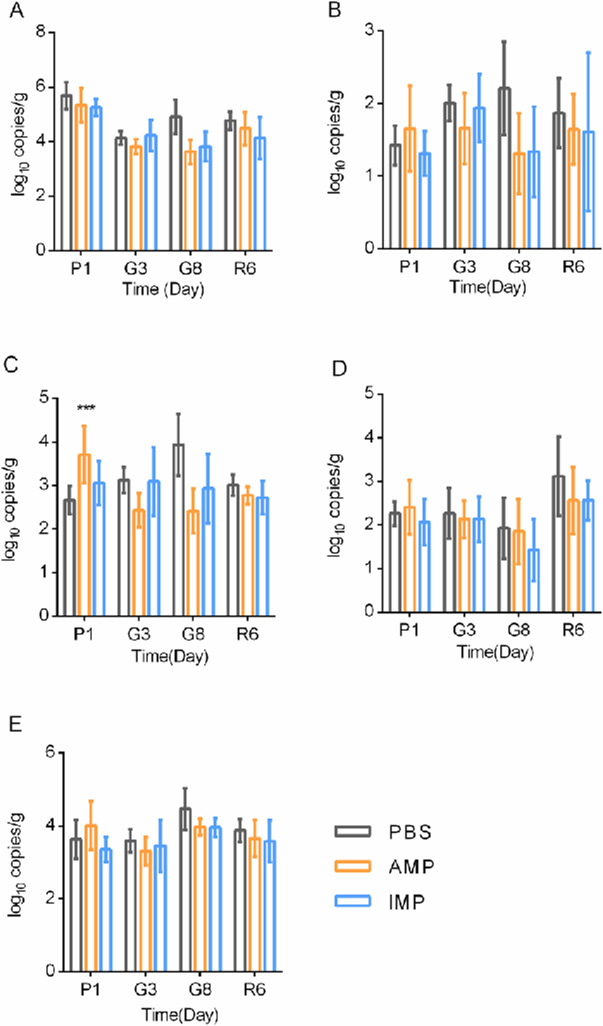
ARG levels in murine fecal samples. (**A**) *bla*_TEM_ levels. (**B**) *bla*_NDM-1_ levels. (**C**) *bla*_IMP-1_ levels. (**D**) *bla*_KPC_ levels. (**E**) *intI1* levels. PBS, PBS-exposed fecal samples; AMP, AMP-exposed fecal samples; IPM, IPM-exposed fecal samples. Statistical analysis was performed with Kruskal-Wallis test. ***, *P* < 0.001.

To examine whether the increase of antibiotic-resistant bacteria in antibiotic-exposed fecal samples was due to an increase in ARG levels, the common representative ARGs for AMP and IPM, *bla*_TEM_, *bla*_NDM-1_, *bla*_IPM-1_, *bla*_KPC_, and an integron-coding gene *intI1* were quantified and compared (Fig. 6). With one-tailed *t*-tests (null hypothesis: AMP or IPM exposure led to increase of ARG levels), no significant increase of these ARGs was found during gavage or recovery. Therefore, increase of antibiotic resistance cannot be supported by increase of ARG levels.

### Persistent antibiotic resistance increase in gastrointestinal tract is due to different mechanisms for Enterobacteriaceae and *Enterococcus*

**Fig 7:**
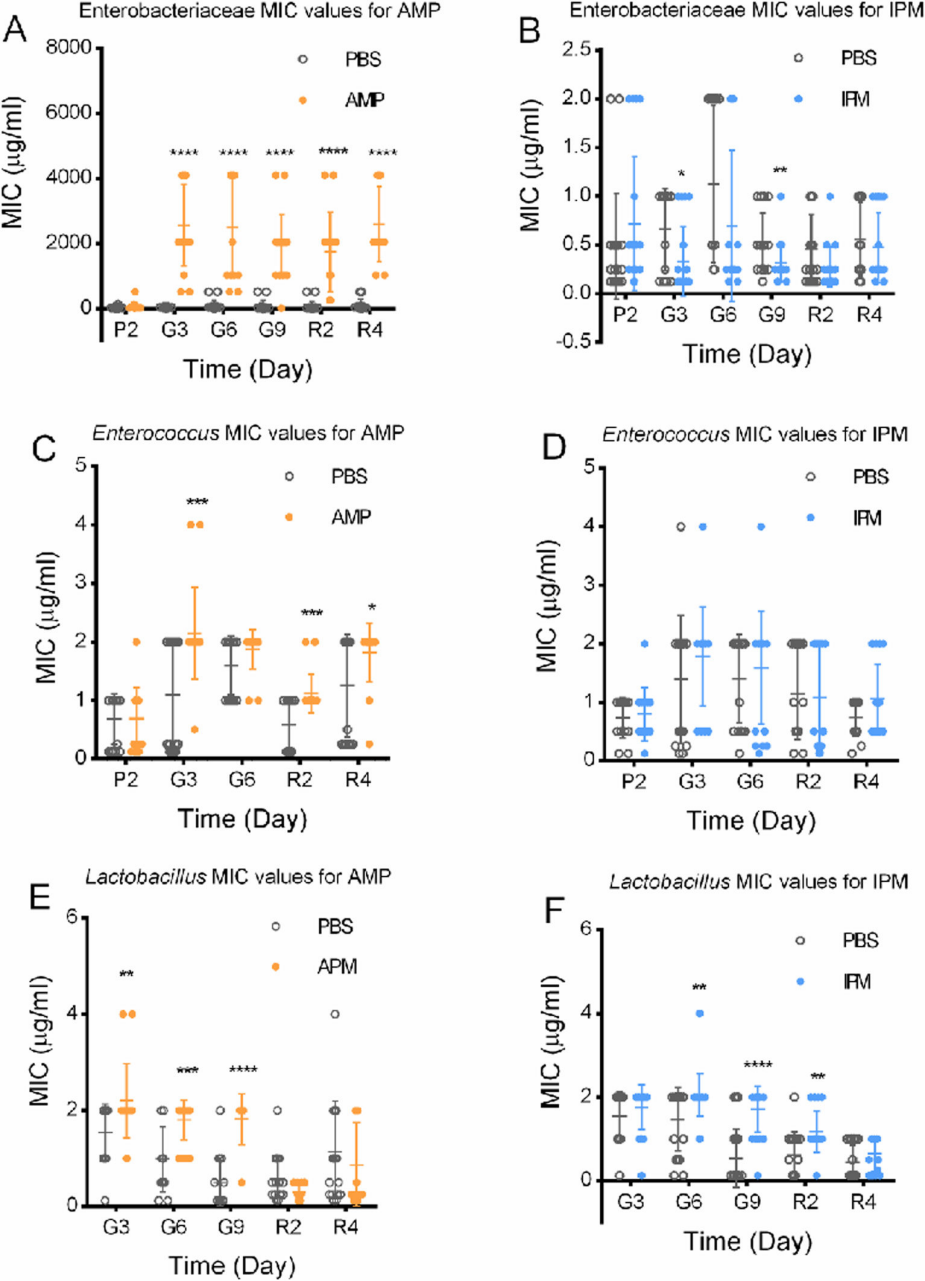
MIC values of isolated strains from murine fecal samples. (**A**) Enterobacteriaceae MIC values for AMP. (**B**) Enterobacteriaceae MIC values for IPM. (**C**) *Enterococcus* MIC values for AMP. (**D**) *Enterococcus* MIC values for IPM. (**E**) *Lactobacillus* MIC values for AMP. (**F**) *Lactobacillus* MIC values for IPM. P2, day 2 before gavage; G3, G6, G9, 3rd, 6th, 9th day of gavage; R2, R4, 2nd and 4th day of recovery. PBS-AMP, AMP-resistant bacteria from PBS-fed mice; AMP-AMP, AMP-resistant bacteria from AMP-fed mice; PBS-IPM, IPM-resistant bacteria from PBS-fed mice; IPM-IPM, IPM-resistant bacteria from IPM-fed mice. At every time point, the Mann-Whitney test was used for significance correction. *, *P* < 0.05; **, *P* < 0.01; ***, *P* < 0.001; ****, *P* < 0.0001. Data for P2 in Panels E and F are not shown because no *Lactobacillus* colonies were recovered in the IPM-gavaged groups.

Enterobacteriaceae, *Enterococcus*, and *Lactobacillus* strains were isolated from antibiotic-free selective media to evaluate their resistance levels by measuring the MIC values for AMP and IPM (Fig. 7). Significant differences were found for Enterobacteriaceae and *Enterococcus* strains. For Enterobacteriaceae, exposure to AMP led to a 50- to 100-fold increase in MIC values for this antibiotic, which persisted through the recovery phase (Fig. 7A), suggesting that the increased MICs account for the increased AMP resistance. Meanwhile, no substantial increase in MICs to IPM was observed for isolated *Enterococcus* strains (Fig. 7D) although the proportion of IPM-resistant *Enterococcus* increased by about five orders of magnitude following gavage. No substantial changes of MICs were found for isolated Enterobacteriaceae to IPM, for isolated *Enterococcus* to AMP, or for isolated *Lactobacillus* strains (Fig. 7B, C, E and F). Although certain MIC values showed significant differences between antibiotic-exposed and PBS-gavaged groups, these changes did not follow a consistent pattern over time and were, therefore, considered incidental rather than indicative of substantial shifts in resistance, consistent with the lack of stimulation of overall antibiotic resistance of these bacteria by exposure to corresponding antibiotics.

**Fig 8:**
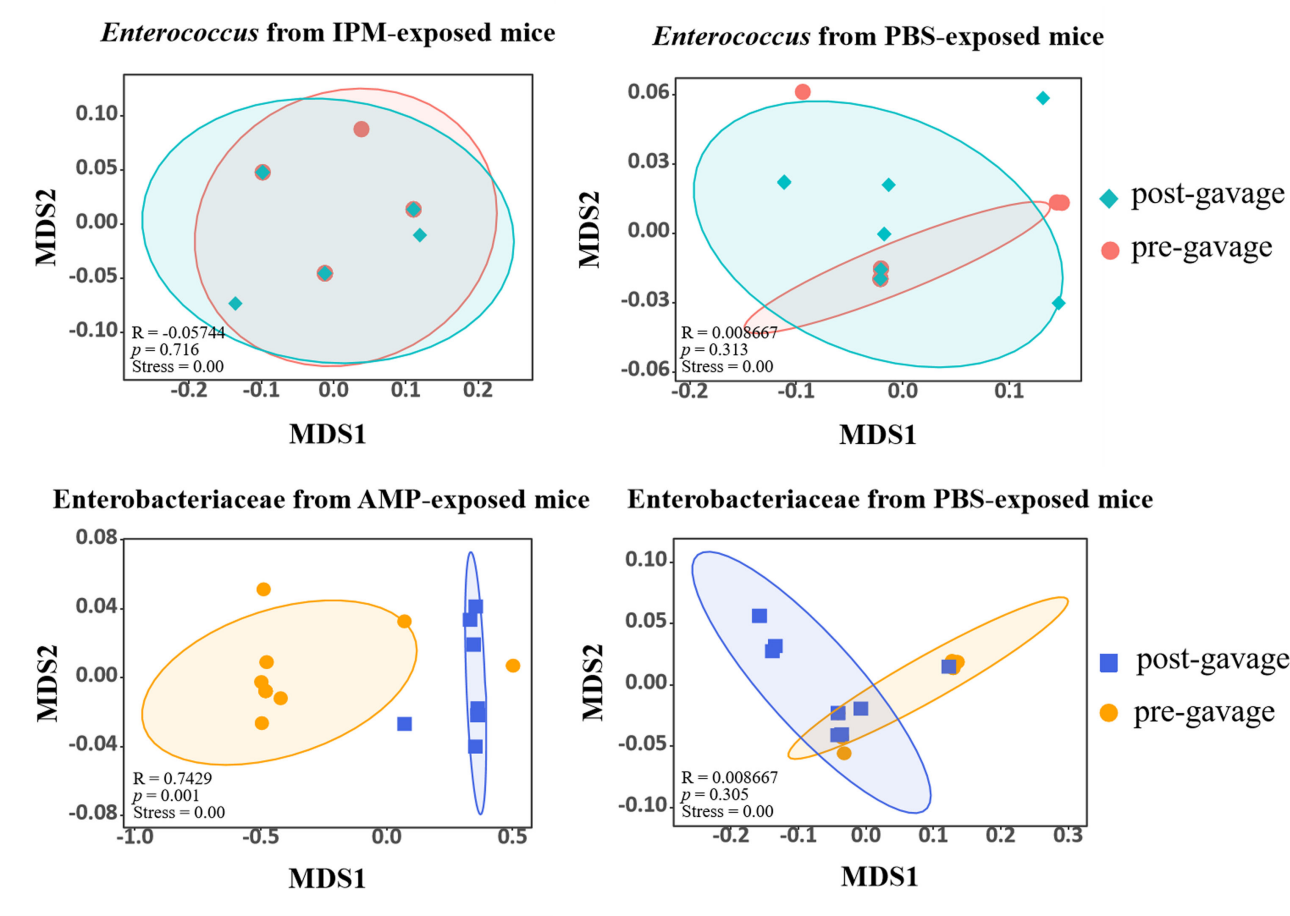
NMDS analysis of Enterobacteriaceae and *Enterococcus* genomes before and after antibiotic exposure.

The different *in vitro* antibiotic resistance phenotypes for Enterobacteriaceae strains after AMP exposure and *Enterococcus* strains after IPM exposure prompted us to hypothesize that the overall increased antibiotic resistance levels are due to different mechanisms. We suspected that AMP exposure led to replacement of gastrointestinal Enterobacteriaceae from AMP-sensitive to AMP-resistant strains, whereas IPM exposure perturbed the physiology of *Enterococcus*, leading to increased resistance without replacing *Enterococcus* strains. To test this hypothesis, we examined whether a strain replacement event occurred after antibiotic exposure by analyzing whole-genome sequences of Enterobacteriaceae and *Enterococcus* strains isolated before (*n* = 10 each) and after (*n* = 10 each) exposure to AMP and IPM, as well as before (*n* = 10 each) and after (*n* = 10 each) exposure to PBS. The Mash distances were calculated between whole-genome sequences as a measure of strain dissimilarity. Strain replacement was determined by assessing whether the genome dissimilarities between strains isolated before and after exposure (between groups) were significantly larger than those between strains isolated before exposure or between strains isolated after exposure (within groups). Data of MASH distance are provided in the supplementary Excel file Supplementary_MASH.xlsx. Exposure to PBS did not lead to strain replacement for either Enterobacteriaceae (ANOSIM, *R* = 0.0087, *P* = 0.305, *n* = 10) or *Enterococcus* (ANOSIM, *R* = 0.0087, *P* = 0.313, *n* = 10). Exposure to IPM did not result in strain replacement for *Enterococcus* either (ANOSIM, *R* = −0.057, *P* = 0.716, *n* = 10), whereas exposure to AMP led to significant and clear strain replacement for Enterobacteriaceae (ANOSIM, *R* = 0.74, *P* = 0.001, *n* = 10). This finding was confirmed by further PCoA showing clearly different grouping of Enterobacteriaceae genomes before and after AMP exposure (Fig. 8). Together, these results support the hypothesis that AMP and IPM exposure increased antibiotic resistance in Enterobacteriaceae and *Enterococcus* through different mechanisms, with AMP exposure specifically causing strain replacement from AMP-sensitive to AMP-resistant Enterobacteriaceae in the gut microbiota.

## DISCUSSION

Maintaining a balanced gut microbiota is critical to sustaining human health. Recent research has clearly revealed the relationship between gut microbiota and a series of medical conditions (49). The bactericidal effects of antibiotics have naturally led to curiosity about how they might affect gut microbiota, and subsequently human health. This has been quite well studied and documented in the past decade, particularly with the maturation of high-throughput sequencing technologies.

Although the sequencing-based metagenomic method has been vastly successful in providing insight into the impact of antibiotics, two flaws accompany the application of this technology: it reports only relative abundances of taxa, and it does not distinguish between live and dead cells. While relative abundances do provide useful information on how microbial community compositions change, it does not count in the changes of total cell amounts. For instance, a decrease in the relative proportion of a bacterium may coincide with an increase in its absolute abundance if the total community size expands, potentially leading to misinterpretation when relying solely on relative data. Although in some cases the relative abundances influence gut microbiota functions, in most scenarios including when assessing bacterial infection potentials, it is the absolute number of viable cells that determines gut microbiota function. Dead cells also carry 16S rDNA genes making them counted in a sequencing-based method. Including these dysfunctional cells will certainly interfere with data analysis and lead to questionable conclusions on the true functions of gut microbiota. We believe, from a functional gut microbiota perspective, considering absolute cell number changes and counting only live cells makes more physiological sense. The present results suggest that absolute abundance data are crucial for interpreting microbiome dynamics, since viable bacteria underpin pathogenicity and resistance, and the observed log-scale increases following antibiotic exposure likely reflect genuine biological responses rather than artifacts of relative quantification. A similar consideration also applies to investigating AMR in gastrointestinal microbiota. Although considering only ARGs and assuming that they represent AMR levels does provide convenience and large data sizes, AMR mechanisms are actually way more complicated than only carrying ARGs (50), and antibiotic resistance phenotypic analysis provides more physiology-relevant, robust conclusions. In addition, qPCR-based assessment of ARGs cannot relate ARGs with bacteria, and a direct association between pathogens and ARGs cannot be concluded. Our findings highlight the critical importance of absolute abundance measurements in microbial ecology. While relative abundance data provide insights into compositional shifts, they may mask substantial changes in total biomass, potentially leading to misleading interpretations. By integrating culture-dependent approaches with DNA-based methods, we were able to capture transient but drastic blooms of opportunistic pathogens under antibiotic pressure—events that would not be apparent from relative metrics alone.

To date, only a handful of studies have considered these aspects when discussing the impact of antibiotics on gut microbiota. Most studies used qPCR methods to evaluate absolute bacterial abundances, which again do not distinguish between live or dead cells (11). Among the several culturing-based studies that considered both absolute bacterial abundances and live cells, O’Sullivan et al. found a decreased *Bifidobacterium* but unaffected *Lactobacillus* and Enterobacteriaceae among human subjects who had recently received antibiotics within one month (51), Rashid et al. found the changes of Gram^+^ and Gram^−^ anaerobic and aerobic bacteria 11 days, 1 month, 2 months, 4 months, and 12 months after antibiotic administration among human subjects (52), Espinosa-Gongora et al. were unable to observe a difference in *E. coli* numbers before and after antibiotic treatment (53), Bernard et al. found decreased Enterobacteriaceae levels immediately after levofloxacin treatment (41). These studies were performed with human subjects, therefore limiting possible data collection and more detailed examination, and more complete observations are yet to be made.

The work reported herein provides a detailed investigation of how antibiotics, more specifically β-lactam AMP and carbapenem IPM, impact on the bacterial levels and resistance in gut microbiota using murine models. Metagenomic analysis was performed to analyze changes in relative abundances, and more importantly, culture- and phenotype-based methods were used to detemine how functional live cells respond to antibiotics in terms of absolute abundance and antibiotic resistance, revealing the impact of antibiotic uptake on gut microbiota functions. Although this study was conducted in a mouse model, the combined use of viable counts, resistance phenotyping, and sequencing is broadly applicable to other host–microbiota systems. This integrative framework provides a generalizable approach for dissecting microbial community responses to perturbations across different biological contexts.

The most striking finding of this work is that absolute cell numbers of live opportunistic pathogen-containing Enterobacteriaceae and *Enterococcus* drastically increased following antibiotic exposure, by as many as six orders of magnitude. This is clear and strong evidence that antibiotic administration leads to blooms of pathogens in the gastrointestinal tracts. This phenomenon can be observed even in cases where resistance to these antibiotics did not increase (IPM resistance for Enterobacteriaceae during IPM exposure, and AMP resistance for *Enterococcus* during AMP exposure). Interestingly, a similar phenomenon for beneficial *Lactobacillus* was absent. The absence of blooms in beneficial *Lactobacillus*, contrasted with the transient increase of opportunistic pathogens, highlights the role of commensals in maintaining colonization resistance. This prompts us to believe that a healthy gut microbiota plays a more pronounced role in resisting pathogens (aka “colonization resistance”).

A significant feature of the total bacterial biomass, represented by 16S rDNA levels and culturable opportunistic pathogens, is that its increase, although significant, is an acute and temporary responses. Even during antibiotic administration, Enterobacteriaceae and *Enterococcus* levels started to drop after peaking at the 3rd or 4th day. This reversibility of apparently detrimental bloom of pathogens suggests the strong resilience of gut microbiota against dysbiosis. We were not aware of this acute response before, probably because it is unlikely to perform investigations on human subjects at this resolution. While it is possible to monitor bacterial levels daily during exposure and recovery with murine models, it is very difficult to do so with human subjects, therefore missing this observation.

While the acute pathogen bloom after antibiotic treatment is temporary, the resistance to antibiotics is not. Although the levels of culturable antibiotic-resistant pathogens nearly returned to pre-exposure levels at the end of recovery, the elevated proportion of resistant pathogens persisted until the end of exposure in the case of IPM-resistant *Enterococcus* and AMP-resistant Enterobacteriaceae, with no sign of decrease. From the quantification of representative ARGs in fecal samples, evaluation of MICs of isolated bacterial strains, and genomic similarity analyses, we believe two different mechanisms underlie this persistence. For the case of AMP-resistant Enterobacteriaceae, a small proportion of the population was AMP-resistant before exposure, and antibiotic treatment quickly selected and enriched this population, making them dominant in gut microbiota. Meanwhile, the competitiveness of AMP-resistant Enterobacteriaceae is not substantially lower than AMP-sensitive Enterobacteriaceae in the absence of antibiotic pressure. Therefore, even when the antibiotic pressure is removed during recovery, the proportion of AMP-resistant Enterobacteriaceae did not decrease. This resistomic ‘scar’ may last a long time, as previously reported (31). For the case of IPM-resistant *Enterococcus*, the key factor for increase of resistance is ‘gavage’ itself, rather than antibiotics. Therefore, we believe a perturbation of environment may lead to transcriptional adaptations that include general stress response, resulting in elevated IPM resistance. This hypothesis is supported by the finding that genomes of isolated *Enterococcus* strains were not significantly different before and after IPM exposure, and IPM resistance levels of isolated strains did not improve, as the strains were sub-cultured to antibiotic-free media prior to MIC determination, and transcriptional adaptations caused by environmental perturbations may have already been “tranquilized.” It needs to be noted that this persistent increase of AMR is not universal for all bacteria or all antibiotics. For instance, the response of AMP-resistance in *Lactobacillus* was also temporary and faded quickly within several days; gavage increased IPM resistance but not AMP resistance of *Enterococcus*.

In conclusion, mouse models were exposed to antibiotics AMP and IPM, and the responses of representative opportunistic pathogens and beneficial bacteria in gut microbiota were investigated. An overall temporally confined increase of bacterial biomass was found following antibiotic exposure. Consumption of antibiotics led to strong, acute, but temporary bloom of opportunistic pathogens with both culturing-based and sequencing-based methods. Meanwhile, antibiotic exposure resulted in a strong and long-lasting increase in antibiotic resistance in Enterobacteriaceae and *Enterococcus,* likely through different mechanisms. This work represents a detailed investigation on how antibiotics impact gut microbiota function in terms of bacterial pathogenesis, with sufficient resolution to capture fine, physiologically relevant functional and phenotypic changes. In doing so, our findings integrate key ecological concepts—such as colonization resistance, resilience to perturbation, and selection for antibiotic resistance—and underscore the broader value of combining culture-dependent and sequencing-based methodologies in microbiome research.

### Conclusion

This work expands the methods in studying the side effects of antibiotic therapy by including culturing methods that measure absolute quantities of live bacteria. The findings revealed that treatment with antibiotics (ampicillin and imipenem) led to a rapid but temporary increase in the absolute abundances of opportunistic pathogens, specifically Enterobacteriaceae and *Enterococcus*, without adversely affecting beneficial bacteria *Lactobacillus*. The changes in antibiotic resistance were significant and persistent, with nearly all Enterobacteriaceae becoming resistant to ampicillin and approximately 10% of *Enterococcus* acquiring resistance to imipenem. Further genomic analysis suggested that the resistance in Enterobacteriaceae was due to strain replacement, while that in *Enterococcus* was not.

## MATERIALS AND METHODS

### Animals and antibiotic exposure

A total of 30 SPF grade 9–15 week old C57/BL6-J mice (male: female = 1:1) were purchased from Beijing Vital River Laboratory Animal Technology co., ltd., and raised in Individual Ventilated Cages (IVC) at 23 ± 1°C, with a light/dark cycle of 12 h/12 h. All feed and water were sterilized.

Mice were fed with gavage daily with ampicillin (AMP, 10 replicates, 0.57 mg/200 µL), imipenem (IPM, 10 replicates, 1.14 mg/200 µL), or 0.2 M phosphate buffer saline (10 replicates, PBS, pH 7.2, 200 µL) daily for 10 days, followed by 10 days of recovery. The dosages of antibiotics correspond to clinical dosages as detailed in the DailyMed database (https://dailymed.nlm.nih.gov/dailymed/index.cfm). Fecal samples were collected daily during exposure and every two days during recovery. Mouse weight was measured every three days.

### Isolation of bacteria

Collected fecal samples were diluted with PBS (1 g feces/15 mL PBS). Isolation of bacteria and calculation of Colony Forming Units (CFUs) were performed with selective media: *Enterococcus* Agar for *Enterococcus*, *Lactobacillus* Selective Agar for *Lactobacillus*, and McConkey Agar for Enterobacteriaceae. *Enterococcus* Agar plates were incubated at 37°C for 36 h, *Lactobacillus* Selective Agar plates were incubated at 37°C for 72 h, and McConkey Agar plates were incubated at 37°C for 12 h. For the counting and isolation of antibiotic resistant bacteria, AMP and IPM was added to plates at the following final concentrations: for *Enterococcus*, AMP 16 µg/mL, IPM 8 µg/mL; for Enterobacteriaceae, AMP 32 µg/mL, IPM 4 µg/mL; for *Lactobacillus*, AMP 8 µg/mL, IPM 0.5 µg/mL. These concentrations were determined following Clinical & Laboratory Standards Institute (CLSI) or EUCAST breakpoints for Minimum Inhibition Concentrations (MICs). Three technical replicates were performed. Sub-culturing of *Enterococcus* and Enterobacteriaceae was performed with Tryptic Soy Broth. Sub-culturing of *Lactobacillus* was performed with *Lactobacillus* Broth Medium. All media for bacteria growth were purchased from Qingdao Hope Bio-Technology Co., ltd., Qingdao, China, and Beijing Aoboxing Bio-Tech Co., ltd., Beijing, China. 16S rDNA sequencing was used to confirm the isolated bacteria on selective media are expected microbes.

### Quantification of antibiotic resistance genes

Total DNA was extracted from fecal samples 2 days before gavage, on the 3rd, 8th day of gavage, and 6th day of recovery, using the lysozyme method. The abundance of *bla*_TEM_, *bla*_NDM-1_, *bla*_IMP-1_, *bla*_KPC_, *intI1*, and 16S rDNA was quantified with the qPCR method similarly to previous protocols (54). Genes were cloned to pMD19-T plasmids for standard curve construction. Primers used for gene cloning and quantification were shown in Table S1. qPCR reactions were performed using a Real-time PCR system (Type StepOnePlus, Applied Biosystems, Waltham, MA, USA) and TB Green Premix Ex Taq (Tli RNaseH Plus) qPCR reagents (Takara Co., ltd., Tokyo, Japan).

### Antibiotic susceptibility tests

Two random colonies were picked for each fecal sample under each condition from antibiotic-free selective plates for each type of bacteria, on the day before gavage, the 3rd, 6th, 9th of gavage, and the 2nd, 4th day of recovery for the determination of MICs. Fecal samples from 10 mice were used for each condition in this experiment, leading two 20 colonies for each condition. Antibiotic susceptibility tests were performed following previously published agar dilution methods following CLSI standards (55). Recommended control strains *E. coli* ATCC25922, *Streptococcus pneumoniae* ATCC49616 and *Enterococcus faecalis* ATCC29212 were used. MICs for each colony were measured twice, and the average was used as the determined MIC value.

### High throughput 16S rDNA amplicon sequencing

High throughput 16S rDNA amplicon sequencing of fecal samples was performed at Novogene Co. ltd (Beijing, China) using Illumina HiSeq 2500 sequencer at the PE250 mode. Fecal samples after the first day of gavage, at the 10th day of gavage, and at the 10th day of recovery were analyzed. Data analysis was performed essentially the same as our previously reported protocol (56). Triplicates for each condition were performed.

### Whole genome sequencing and analysis

Ten *Enterococcus* strains were randomly picked before and after gavage of imipenem and PBS (a total of 40 strains), and 10 Enterobacteriaceae strains were randomly picked before and after gavage of ampicillin and PBS (a total of 40 strains). Genomic DNA was extracted from the 80 strains and subject to second-generation high-throughput second generation sequencing (BGI DNBSEQ-T7 at PE150 mode). Genome assembly was performed using SPAdes 3.15.5. Quality control was done with QUAST 5.2.0, BUSCO 5.2.2, and CheckM 1.1.2. Only contigs above 500 bp were retained. Taxonomic identification was performed with GTDB-TK 2.3.0. Distances between genomes were calculated with Mash 2.3. Anosim on R platform was used to determine changes of bacterial population before and after gavage. PCoA analysis was performed with the vegan package on R platform. For the naming of strains, strain numbers starting EPA indicate Enterobacteriaceae strains isolated before gavage of ampicillin, strain numbers starting EPP indicate Enterobacteriaceae strains isolated before gavage of PBS, strain numbers starting ERA indicate Enterobacteriaceae strains isolated after gavage of ampicillin, strain numbers starting ERP indicate Enterobacteriaceae strains isolated after gavage of PBS, strain numbers starting CPI indicate *Enterococcus* strains isolated before gavage of imipenem, strain numbers starting CPP indicate *Enterococcus* strains isolated before gavage of PBS, strain numbers starting CRI indicate *Enterococcus* strains isolated after gavage of imipenem, strain numbers starting CRP indicate *Enterococcus* strains isolated after gavage of PBS.

### Statistics

Unless otherwise specified, comparison of data was performed with two-tailed unpaired *t*-tests. All statistical analyses were performed using GraphPad Prism 9.4.1 or Jamovi 2.3.21. *P* < 0.05 was considered statistically significant.

## Data Availability

The 16S amplicon data can be found at Genbank under BioProject PRJNA982033. The whole genome sequencing data can be found at Genbank under BioProject PRJNA981323.

## SUPPLEMENTAL MATERIAL

Data Set S1 spectrum.01104-25-s0001.xlsx MASH distance data.

Supplemental Material spectrum.01104-25-s0002.docx Figures S1 to S4, Table S1, and supplemental methods.

ASM does not own the copyrights to Supplemental Material that may be linked to, or accessed through, an article. The authors have granted ASM a non-exclusive, world-wide license to publish the Supplemental Material files. Please contact the corresponding author directly for reuse.
