# Supplementary material for: The expansion of culturable opportunistic pathogens and antibiotic resistance in mouse gut following antibiotic exposure
Source: Microbiol Spectr. 2025 Dec 5;14(1):e01104-25. doi: 10.1128/spectrum.01104-25 (PMC12772332; doi:10.1128/spectrum.01104-25)
Supplement: Supplemental Material — Figures S1 to S4, Table S1, and supplemental methods. [file spectrum.01104-25-s0002.docx]

**Supplementary materials**

**
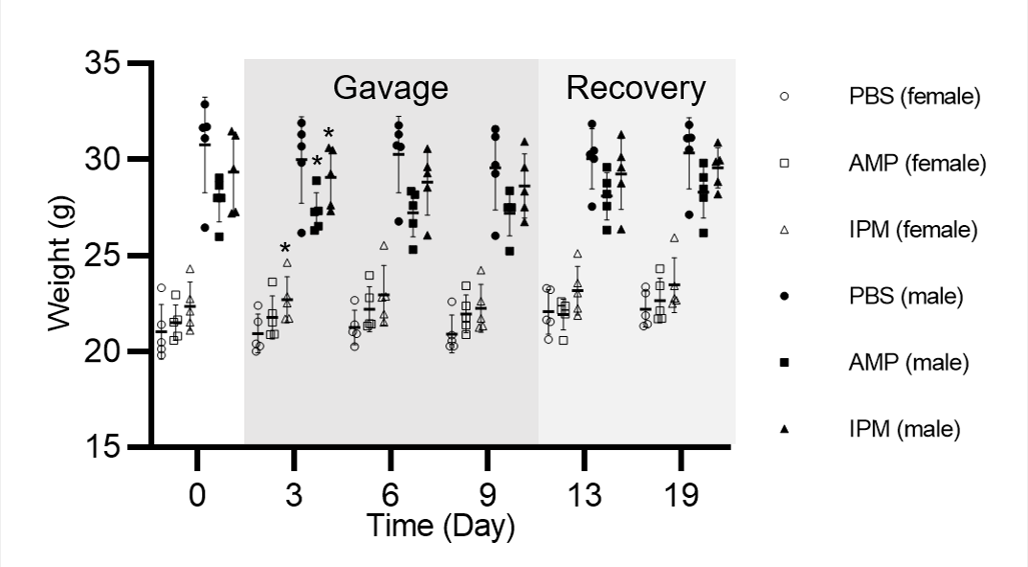
**

**Figure S1. Mouse weights.** Day 0 represents mouse weight before gavage. *, *p*<0.05.

**
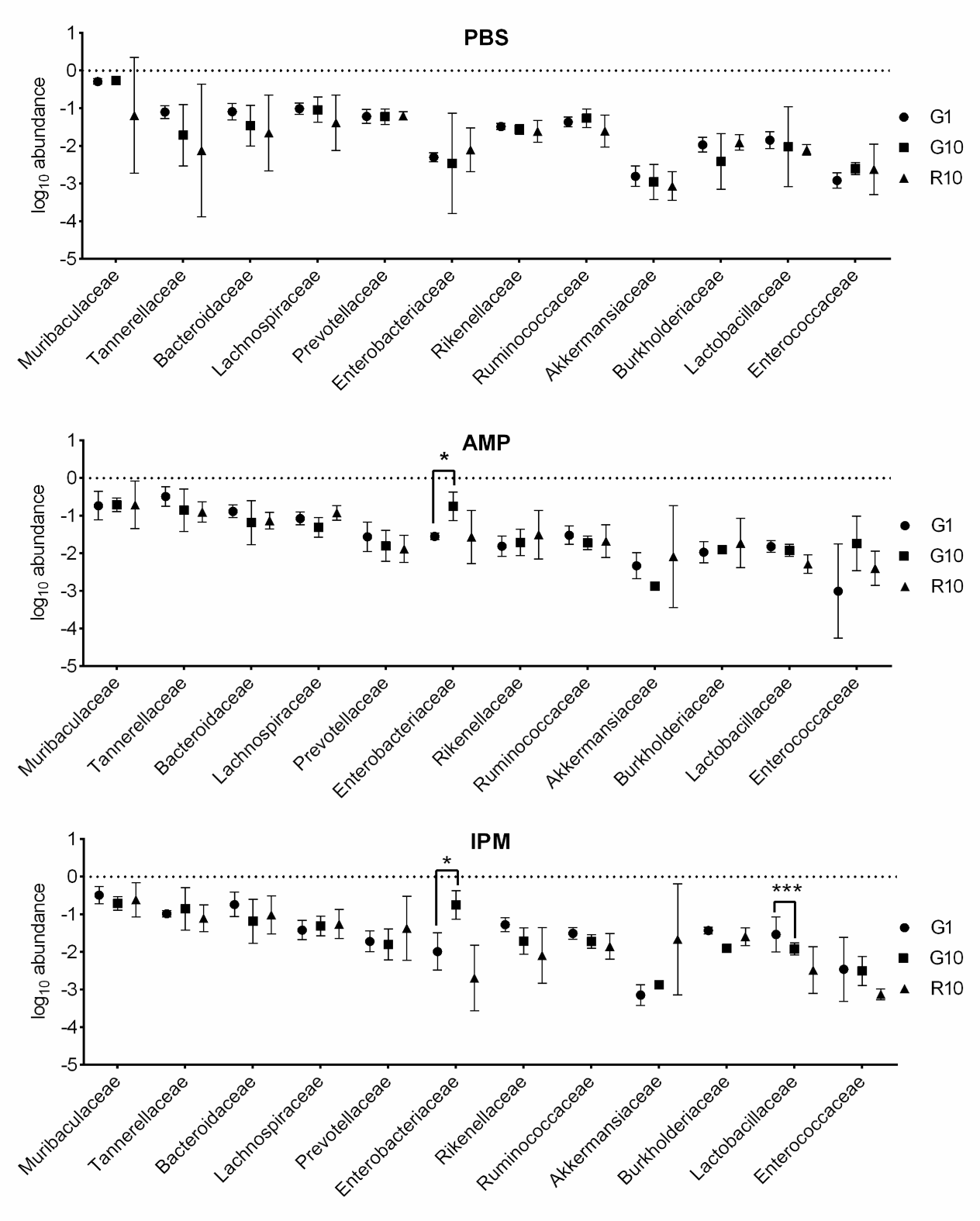
**

**Figure S2. Comparison of murine fecal microbiomes at the family level.** Enterococcaceae and other families with an average abundance of over 1% were selected. G1, first day after gavage; G10, 10^th^ day after gavage; R10, 10^th^ day of recovery. *, *p*<0.05; **, *p*<0.01. Error bar, standard deviation.

**
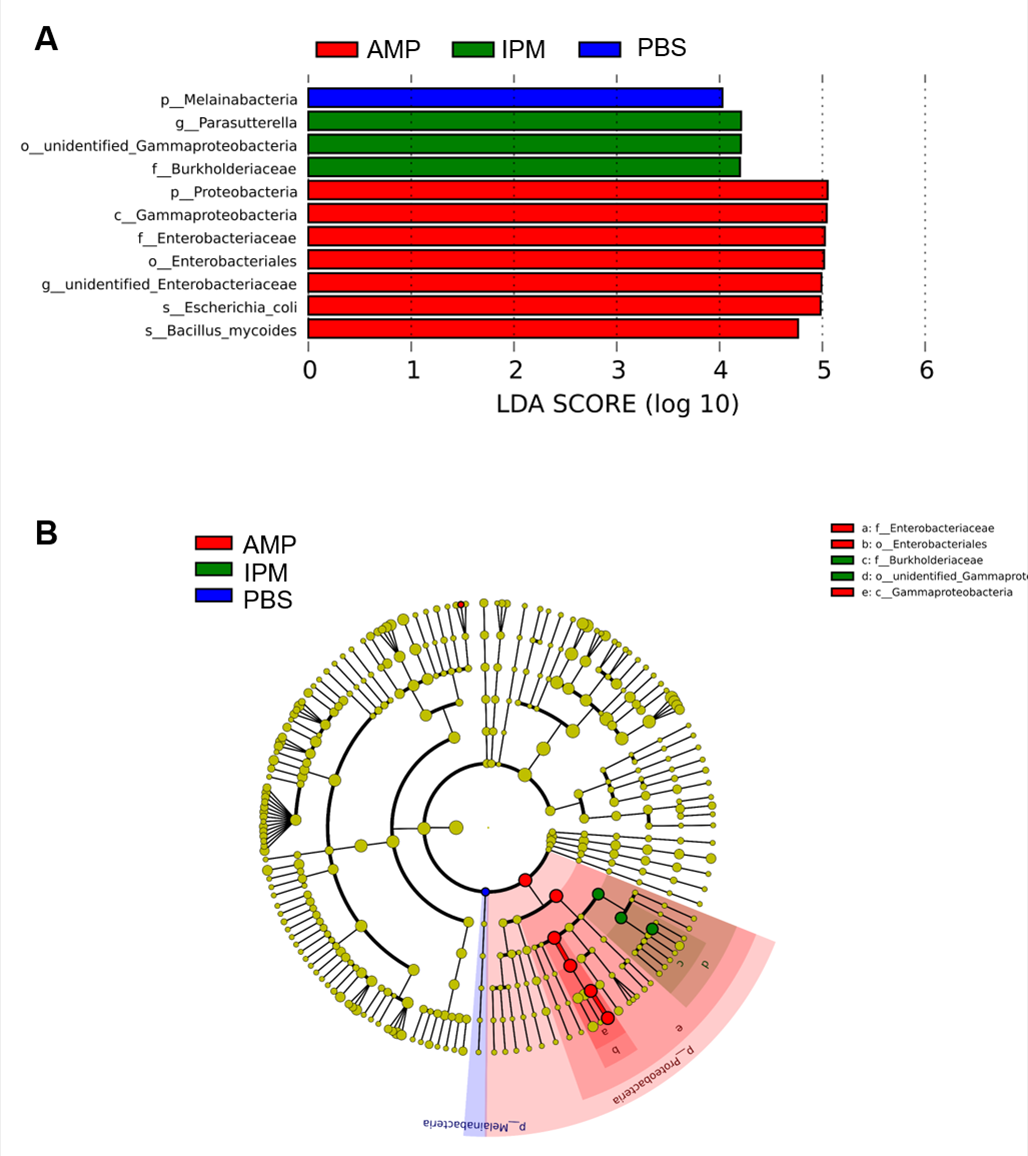
**

**Figure S3. LefSe analysis of PBS- or antibiotic-exposed murine fecal microbiomes.** Panel A, LDA scores; Panel B, cladogram.

**
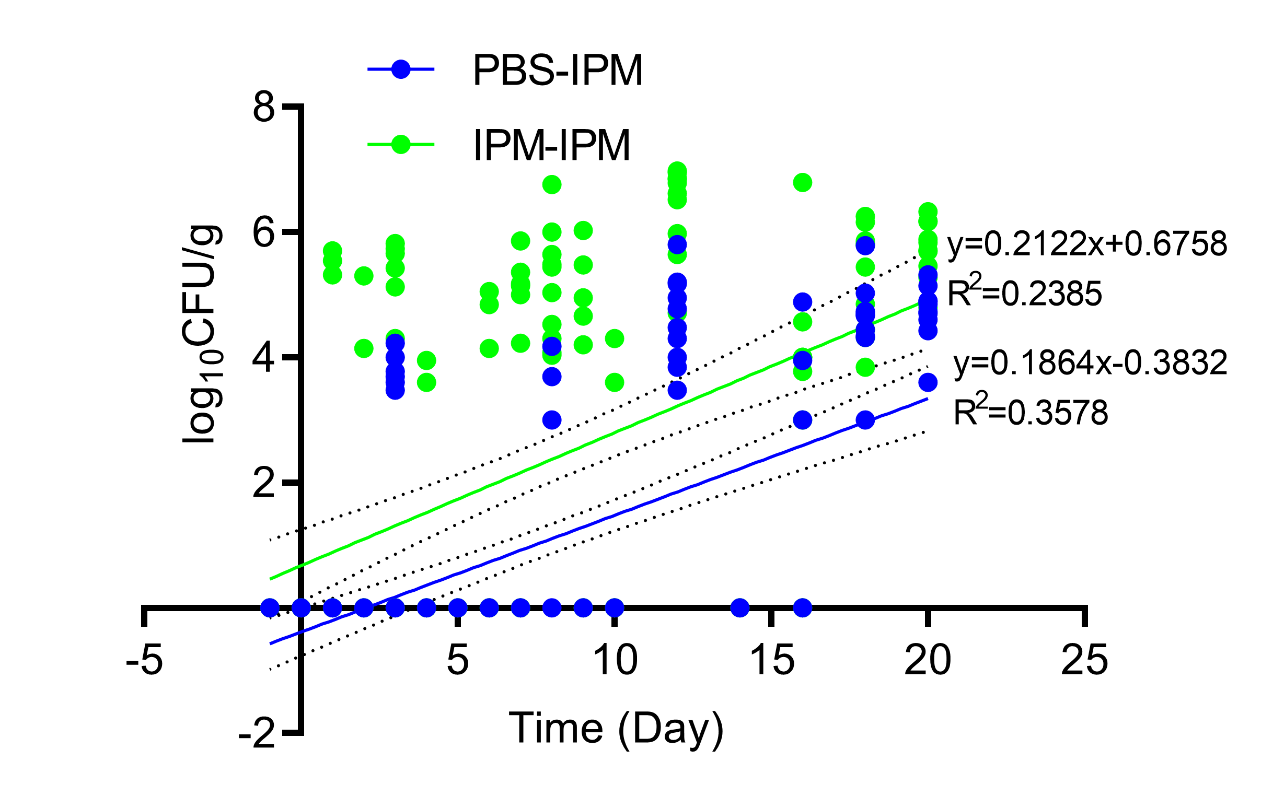
**

**Figure S4. Linear regression of IPM-resistant *Enterococcus* increase.** PBS-IPM, IPM-resistant bacteria in fecal samples from PBS-exposed mice; IPM-IPM, IPM-resistant bacteria in fecal samples from IPM-exposed mice. Days are counted starting from the first day of gavage. Negative values indicate pre-gavage days.

**Table S1. Primers used in this work.**

| Targeted gene | Sequence (5’-3’) | Fragment length (bp) | References |
| --- | --- | --- | --- |
| 16S rDNA (Forward) | CCCAGATGGGATTAGCTTGT | 106 | (1) |
| 16S rDNA (Reverse) | TCTGGACCGTGTCTCAGTTC |  |  |
| *intI1* (Forward) | GCCTTGATGTTACCCGAGAG | 196 | (2) |
| *intI1* (Reverse) | GATCGGTCGAATGCGTGT |  |  |
| *bla*_TEM_ (Forward) | GCGCCAACTTACTTCTGACAACG | 247 | (3) |
| *bla*_TEM_ (Reverse) | CTTTATCCGCCTCCATCCAGTCTA |  |  |
| *bla*_IMP-1_ (Forward) | GGCTTAATTCTCGATCTATCCC | 114 | (4) |
| *bla*_IMP-1_ (Reverse) | CTAGCCAATAGTTAACTCCGC |  |  |
| *bla*_NDM-1_ (Forward) | GCCCAGATCCTCAACTGGAT | 135 | This work |
| *bla*_NDM-1_ (Reverse) | CGCATTGGCATAAGTCGCAA |  |  |
| *bla*_KPC_ (Reverse) | TTACGGCAAAAATGCGCTGG | 201 | This work |
| *bla*_KPC_ (Forward) | TCCAGACGGAACGTGGTATC |  |  |

**Supplementary_MASH.xlsx**

CPICRI, Mash distances between *Enterococcus* isolates before and after IPM gavage.

CPPCRP, Mash distances between *Enterococcus* isolates before and after PBS gavage.

EPAERA, Mash distances between Enterobacteriaceae isolates before and after AMP gavage.

EPPERP, Mash distances between Enterobacteriaceae isolates before and after PBS gavage.

**References**

1. Kim JY, Lee JL. 2014. Multipurpose assessment for the quantification of *Vibrio* spp. and total bacteria in fish and seawater using multiplex real-time polymerase chain reaction. J Sci Food Agric 94:2807–2817.

2. Barraud O, Baclet MC, Denis F, Ploy MC. 2010. Quantitative multiplex real-time PCR for detecting class 1, 2 and 3 integrons. J Antimicrob Chemother 65:1642–1645.

3. Xi C, Zhang Y, Marrs CF, Ye W, Simon C, Foxman B, Nriagu J. 2009. Prevalence of antibiotic resistance in drinking water treatment and distribution systems. Appl Environ Microbiol 75:5714–5718.

4. Mentasti M, Prime K, Sands K, Khan S, Wootton M. 2019. Rapid detection of IMP, NDM, VIM, KPC and OXA-48-like carbapenemases from Enterobacteriales and Gram-negative non-fermenter bacteria by real-time PCR and melt-curve analysis. Eur J Clin Microbiol Infect Dis 38:2029–2036.
